# The risk of water, sanitation and hygiene on diarrhea-related infant mortality in eastern Ethiopia: a population-based nested case-control

**DOI:** 10.1186/s12889-022-12735-7

**Published:** 2022-02-18

**Authors:** Samuel Mebrahtom, Alemayehu Worku, Daniel J. Gage

**Affiliations:** 1 Ethiopian Institute of Water Resources, Addis Ababa University, Addis Ababa, Ethiopia; 2 School of Public Health, College of Health Sciences, Addis Ababa University, Addis Ababa, Ethiopia; 3 Department of Molecular and Cell Biology, University of Connecticut, Storrs, CT 06269 USA

**Keywords:** Diarrhea-related infant mortality, Risk factors, Water, Sanitation and hygiene

## Abstract

**Background:**

Diarrhea is still appeared to be as one of the leading global killers and disability-adjusted life-years lost, particularly in the infant and children. As per WHO, about 88% of diarrhea-related deaths are attributable to unsafe water, inadequate sanitation and insufficient hygiene, mainly in developing world. Thus, the main objective of this study was to find out the risk of such factors that contribute for diarrhea-related infant mortality in Eastern Ethiopia.

**Methods:**

This study employed community based unmatched nested case-control study design in Eastern Ethiopia. The cases were infants who died from diarrheal disease while controls were those who survived their first year of life from September, 2016 to August, 2018. A total of 305 study subjects (61 cases and 244 controls) were included in the study. Infants dying from diarrhea were compared to four neighborhood controls in terms of several risk components of Water, Sanitation and Hygiene. Data were collected from mothers/care takers of infants using pre-tested structured questionnaires, and entered onto CSpro version 5.1 and transform to SPSS version 23 to analyzed potential risk factors.

**Findings:**

Finding of this study revealed that the risk factors that found to be significantly associated with infant death from diarrhoea after adjustment for confounding variables included the age of mother with < 20 years old (*P* = 0.009, AOR: 0.01, 95% CI: 0.01, 0.47), unsafe drinking water storage (*P* = 0.013, AOR: 0.4, 95% CI: 0.18, 0.81), infants in households without point-of-use water treatment practices (*P* = 0.004, AOR: 0.21, 95% CI: 0.08, 0.61), households with unimproved sanitation (*P* = 0.050, AOR: 0.36, 95% CI: 0.13, 1.00), unsafe disposing of child feces (*P* = 0.014, AOR: 0.34, 95% CI: 0.15, 0.81), and improper management of solid waste (*P* = 0.003, AOR: 0.29, 95% CI: 0.13, 0.66). These exposure factors had lower risk for the contribution of infants dying from diarrhoea than those with their reference group in the study area. However, infants in households with improper management of liquid waste management showed strongly significant association which had three times more likely to occur diarrhea-related infant death (*P* = 0.010, AOR: 3.43, 95% CI: 1.34, 8.76). Similarly, infants whose mother/caretaker practiced hand washing with less critical time (one-two occasions) had three times greater risk to infant death from diarrhea than those who had practice more than three critical times of hand washing (*P* = 0.027, AOR: 3.04, 95% CI: 1.13, 8.17).

**Conclusion:**

This study suggests that infants in households with improper management of liquid waste and hand washing practices with fewer occasions (one-two critical time) are a greater risk of getting a diarrhea-related infant death. Therefore, efforts should be made to ensure intervention taking such risk factors into consideration, typically in the infantile period.

## Background

Diarrhea is a major cause of morbidity and mortality, in particular, among infants and children worldwide [1]. It is still appeared to be as one of the leading global killers [2, 3] and disability-adjusted life-years lost [4, 5]. According to the World Health Organization, more than half a million of diarrheal-related deaths reported among under five children each year in worldwide [6, 7]. In 2019, diarrhea was responsible for about 7.4% of all global causes of deaths to children < 5 years of age [8]. Around 90% of all diarrhea-associated deaths occur in children under five years of age, particularly in low-and-middle income countries [9, 10].

Despite substantial reduction of the total annual number of diarrheal-related death is observed in the world among children < 5 years over the period 1990 and 2017, the number of death remained highest in some of the world’s developing countries [11]. Evidences indicated that south Asia and sub-Saharan Africa were among the highest death rates areas, where 78% of childhood diarrheal deaths occurred [12]. From all deaths, the top five countries where the most frequent diarrhea deaths occurred include: Nigeria, India, Pakistan, Democratic Republic of Congo and Ethiopia [12, 13]. While the majority of diarrhea cases occur in developing countries, developed nations also experience a considerable burden from diarrhea [14].

Diarrhea has been shown to be one of the main causes of infant mortality in Ethiopia [8, 15], and its burden is still a serious concern [16–19]. Although the under five deaths due to diarrhea decreased from 16 to 8% between 2000 and 2016, the reduction trend of diarrheal death in this country observed to be unsteady [15]. Diarrheal disease is the fourth leading cause of infant death in Ethiopia, responsible for death rate of 136.6 deaths per 100,000 populations by the year 2019 [8]. Evidences have shown that the link between diarrheal death and the risk of unsafe water supply, inadequate sanitation, and insufficient hygiene practices are very much strong, contributing for 88% of diarrhea-related deaths [20–22]. In Ethiopia, an estimated 35.1 and 93.7% of households lack access to improved water supply and sanitation respectively and yet, rate of handwashing with soap remain low (17.6%) [16]. The regions resided in eastern part of Ethiopia have been known as one of the area where diarrhea morbidity and mortality are observed [16]. The medical report suggests that diarrhea is among the top ten morbidity cases in the districts found in Eastern Ethiopia.

According to WHO, diarrhea is commonly defined as the passage of loose or watery stools occurring three or more times in a 24-h period, and causes death by depleting body fluids resulting in profound dehydration [23]. It can be easily treatable and preventable [22–24]. Most of diarrhoea-related deaths are appeared in small children [25, 26]. Infants (< 1 year of age) is being placed at the highest death from diarrhea among children under 5 years of age [14, 27]. Diarrhea can have a harmful impact on childhood growth and cognitive development [28]. Hence, paying a particular attention to this age group will turn down a marked effect on infant mortality. There are multiple risk factors that likely to be responsible for the cause of childhood diarrhea-related mortality. Along with biological and social factors, environmental factors such as deficient in Water supply, Sanitation and Hygiene are one of the main risk factors that contribute for diarrheal death of infants [29–31], and also the leading risk factor identified in the world [23, 25, 32].

Publications that has examined on diarrheal death related risk factors were rare [30]. Indeed, many studies were conducted on under five predominantly in diarrheal morbidity [32], and studies on risk factors that makes diarrhea-related death more likely to happen particularly on infants (< 1 year of age group) were very much limited and has not yet been analyzed in the study area. Hence, there is need such extensive study which helps to develop better understanding for selecting strategies in reducing the death due to diarrhea. Accordingly, the main objective of this particular study was to identify the risk of drinking water supply, sanitation and hygiene components that contribute to diarrhea-related infant mortality in Eastern Ethiopia. This study assumed to be filled the existing gap and enable policy and decision makers to develop preventive strategy that can help infant survival against diarrhea.

## Materials and methods

### Study setting

This study was conducted in six randomly selected districts in West Hararghe administrative zone of Oromia region and Zone 3 of Afar region, which are situated in Eastern part of Ethiopia. The districts include Chiro, Mieso, Gemechis and Tullo districts of West Hararghe administrative zone of Oromia while Amibara and Awash Fentale districts that comprised in zone 3 administration of Afar region. Based on the 2007 National Population and Housing Census of Ethiopia [33], the projected population estimate for the year 2019 to each districts; Chiro (243,151), Mieso (191,978), Gemechis (263,615), Tullo (212,234), Amibara (85,964) and Awash Fentale (40,448). Of the total population, 3.4% are under the age of one year in the national context [33]. The study area has two main climate seasons - a dry season and a rainy season: The dry season ranges from October to February while the rainy seasons have two periods – the period from June to September is the main rainy season and some rainy weather period usually from March to May [34]. As of 2019, the health facilities available to each districts; Chiro (One hospital, 7 health centres and 39 health posts), Meiso (One hospital, 3 health centres and 33 health posts), Gemechis (6 health centres and 36 health posts), Tullo (8 health centres and 30 health posts), Amibara (one hospital, 3 health centres and 13 health posts) and Awash Fentale (2 health centres and 11 health posts). As a common, the most frequently cases observed in the districts are Acute upper respiratory tract infection, Lower respiratory infections such as pneumonia and bronchitis, Diarrheal diseases, Malaria, Urinary tract infections and skin infections. Due to inconsistency of secondary data, access coverage to improved water supply, sanitation and good hygiene practices is challenging in each districts. However, as per the expert’s opinion, most of the districts population have inadequate access to improved drinking water and sanitation, and insufficient hygienic practices were still a common problem.

### Study design

The study employed community based nested unmatched case-control study, which was nested from longitudinal survey conducted to identify cause of infant mortality from September, 2016 to August, 2018 in Eastern Ethiopia.

### Study population

The study population (mother-infant pair) consisted of cases and controls drawn from the longitudinal survey conducted in the randomly selected study area. Cases were infants who died from diarrheal disease (all 61 cases that have been identified in the study area during the study period were included) while controls were those who survived their first year of life.

### Sample size determination

The sample size was computed using Openepi version 3.03a, and calculated with the sample size for unmatched case control study. The sample size was calculated for each variable taking the result of the study conducted previously that would allow to taking the largest sample size. Accordingly, the sample size was determined based on the assumption that the proportion of latrine of the household as a risk factor for diarrhea-related infant death among the household of controls to be 15.4 and 5.93% for cases [30], two-sided significance level (1-alpha) = 95%, Power (1-beta, % chance of detecting) = 90, Ratio of Unexposed to Exposed in sample = 1:4, to detect an odds ratio of 0.29. Thus, the minimum sample size required for the study estimated to be 290 (58 cases and 232 controls). Because of many reasons of uncertainty and possible non-response, 10% were added, thus the final sample size resulted as 319 (64 cases and 255 controls).

### Sampling technique

The sampling techniques for this particular study exploit all diarrheal deaths of infants, which were selected as cases while density sampling used for the control groups. The entire cases (infant died from diarrhea) that ascertained by electronics verbal autopsy were directly taken from the longitudinal survey. Infants who had survive their first year were eligible for selection as control group, which were randomly selected from the adjacent area linked to that of the infants died due to diarrhea. For each case, four neighborhood controls were randomly selected from the longitudinal survey database and compared in terms of socio-demographic and several risk components of Water, Sanitation and Hygiene.

### Data collection instrument and methods

Data were collected by trained data collectors using structured and pre-tested questionnaire and conducted under closely overseen by supervisors. The questionnaire was prepared based on WHO, UNICEF and national standards as well as adopted from relevant literatures [35, 36]. This questionnaire was first developed in English, then translated into both “Amharic” and “Afan Oromoo”, and back translated into English to ensure its meaning of questions is retained and checked consistency. The respondents were mothers or primary caretaker of the infants (< 1 year of age).

### Data quality control

Data quality was assured by controlling both random and systemic error. Data quality was maintained through properly designed data collection tool, and data collectors as well as supervisors recruited with the relevant educational background and language proficiency. Training was held and pre-test carried out in a community with similar characteristics. The data collection procedures developed and the collected data were reviewed by principal investigator. Any identified errors were discussed and immediate measure has been taken.

### Data management and analysis

The collected data were entered into CSPro version 6.1 then transformed to SPSS version 23 for analysis. Descriptive statistics such as frequency distribution and cross tabulation were made to summarize the study variables. Both bivariate and multivariable conditional logistic regression was used to estimate crude and adjusted odds ratios with 95% confidence intervals for the association between risk factors and diarrhea-related infant death. Bivariate conditional logistic regression for each variable was analyzed and any risk factors that showed marked association (*p*-value < 0.25) were considered as candidate for multivariable logistic regression. The Hosmer-Lemeshow goodness-of-fit test was checked and indicated as this model is valid seeing that the *p*-value is greater than 0.05. Variables that resulted *P* value < 0.05 in the multivariable logistic regression were affirmed as significantly associated with the outcome variable (diarrhea-related infant death).

### Operational definitions

#### Water accessible

People access to 25 l per capita per day within 1 km radius from improved water supply sources for rural community [36].

#### Households adequate water treatment at point-of-use

Boiling, add bleach/chlorine, water filter (ceramic, sand, composite) and solar disinfection [35].

#### Safe water storage

Water stored in plastic, clay or metal pot narrow mouth (usually diameter of 3 cm or less), have a lid or secured cover and a tap (spigot), cleaned and kept cover [37].

#### Improved sanitation

Flush/pour toilet, Ventilated Improved Pit (VIP) latrine, simple pit latrine with slab (slab that can be cleaned), composting toilet [35].

#### Access to hand washing facilities near to latrine

Presence of hand washing station within 3 m of the latrine with water and soap/substitute.

#### Critical time of hand washing

Hand washing with soap or substitute at critical times (the most recommended occasions): after using latrine, after cleaning child bottom, before preparing food, before feeding child, before breastfeeding.

#### Safe disposal of children’s faeces

Child used toilet/latrine, put/rinsed faeces into the toilet/ latrine and buried the faeces [35].

#### Proper solid waste management

Households dispose their wastes to waste collection tank, provide to private waste collection groups, buried and/or burn, and composting.

#### Proper liquid waste management

Households dispose their liquid wastes through infiltrate to the ground, cesspool (a pit dug in the ground to receive liquid waste) and dumping to municipal disposal sites.

## Results

### General characteristics of study subjects and respondents

A total of 305 study subjects (61 cases and 244 controls) were included in the study, which yields the non-response rate 4.7 and 4.3% for case and control, respectively. Among these studied subjects, 61 were infants who died as the result of diarrheal disease (cases),and 244 were those who survived their first year of life (controls),which was nested in a longitudinal survey database (from September 2016 to August 2018) residing in Eastern Ethiopia.

### Socio-demographic characteristics associated with infant diarrheal death

Of the Interviewed infants’ mothers/caretakers, the mean age (± SD) of the respondent among cases (infant who died due to diarrhea) was 26.8 (±3.9) years old and 27.8 (±4.5) for controls (infant who survived their first year of life). The majority of the infants’ mothers of the cases fall within the youth age group of 20-34 (90.2%), while it was (82.0%) for the controls. About 44.3% of cases and 48.4% of controls had history of having borne two to four viable offspring (Parity). The mean (± SD) family size of households with infants in the cases was 4.70 (±1.98) and 4.99 (±1.96) for controls. Majority of the study participants were married at 96.7% of cases and 98.0% of controls. Oromo ethnic group comprises the largest proportion of the study subjects (90.2% of cases and 87.3% of controls). Muslim followers were larger in the study participants at 98.4% for cases and 88.5% for the control group. The majority of the respondents (88.5% of cases and 74.2% of controls) were not educated. Likewise, most of the spouse of the cases (78.7%) was uneducated as compared with the controls (65.2%). Almost equal proportion of the cases (90.2%) and the controls (90.6%) were housewives by occupation. Spouse’s occupational status between the two-study groups indicated that about (90.2%) cases and (85.7%) controls were found to be farmers/own farm labor. High proportion of controls (50.8%) compared with that of cases (45.9%) had an average household monthly income of more than and equal to 570 ETB.

The bivariate analysis between socio-demographic characteristics and diarrhea-associated infant death indicated that mothers and spouse’s level of education were significantly associated with infant’s diarrheal death. In this analysis, it was estimated that the infants whose mothers were not educated had less likely for risk of infant death as a result of diarrhea than those whose mothers had reached at some level of schooling (*P* = 0.021, COR: 0.37, 95% CI: 0.16, 0.86). Likewise, the death of infants due to diarrhea was less likely to occur for the uneducated spouse than educated (*P* = 0.046, COR: 0.51, 95% CI: 0.26, 0.98) (see Table 1).

**Table 1:** Frequency distribution and bivariate analysis of socio-demographic characteristics with diarrhea cases and controls in Eastern Ethiopia, 2016-18

Socio-demographic Characteristics	Case		Control		Crude Odds Ratio (95% CI)	*P*-value
	n	%	n	%		
Age of the mother						
< 20 Years old	5	8.2	18	7.3	0.14 (0.02, 1.05)	0.056
20-34 Years old	55	90.2	200	82.0	1.01 (0.36, 2.84)	0.985
> 35 years old	1	1.6	26	10.7	1	–
Parity						
1st	22	36.0	72	29.5	0.97 (0.46, 2.06)	0.939
2nd-4th	27	44.3	118	48.4	1.34 (0.71, 2.52)	0.372
> 5	12	19.7	54	22.1	1	–
Household Family Size						
> 5	18	29.5	91	37.3	1.42 (0.77, 2.61)	0.258
< 5	43	70.5	153	62.7	1	–
Maternal Marital Status						
Married	59	96.7	239	98.0	1.62 (0.31, 8.56)	0.570
Unmarried	2	3.3	5	2.0	1	–
Ethnicity						
Oromo	55	90.2	213	87.3	0.75 (0.29, 1.89)	0.540
Others	6	9.8	31	12.7	1	–
Religion						
Muslim	60	98.4	216	88.5	5.65 (0.75, 42.9)	0.094
Christian	1	1.6	28	11.5	1	–
Mother’s level of education						
No education	54	88.5	181	74.2	0.37 (0.16, 0.86)*	0.021
Educated	7	11.5	63	25.8	1	–
Spouse’s level of education						
No education	48	78.7	159	65.2	0.51 (0.26, 0.98)*	0.046
Educated	13	21.3	85	34.8	1	–
Mother’s Occupation						
Housewife	55	90.2	221	90.6	1.05 (0.41, 2.69)	0.922
Others	6	9.8	23	9.4	1	–
Spouse’s Occupation						
Farmer/own farm labor	55	90.2	209	85.7	0.65 (0.26, 1.63)	0.359
Others	6	9.8	35	14.3	1	–
Households Average Monthly Income (ETB)						
<570ETB	33	54.1	120	49.2	0.82 (0.47, 1.44)	0.492
>570ETB	28	45.9	124	50.8	1	–

*Risk factors significantly associated at *p*-value ≤ 0.05

### Environmental variables (water supply, sanitation and hygiene) associated with risk of infant death due to diarrhea

The distribution of cases and controls as well as bivariate analysis in the different categories of Water supply, Sanitation and Hygiene presented as follows:

### Risk of access and use of water supply associated with infant’s diarrheal death

Almost equal proportion of case (78.7%) and control (78.3%) group of infant’s households were used improved water sources. The household’s time to access water source resulted with 30 min or less (65.6% of case and 62.7% of control). About 85.2% of cases and 82.0% controls fetched water within 1 km radius from their dwelling. Most of the infants in the households with less than 25 l water consumption per capita per day among case and control group appears to be 88.5 and 75.4%, respectively. The vast majority of cases (91.8%) and controls (79.1%) among infants in the households found with water inaccessibility (i.e households not access to at least 25 l per capita per day within a distance up to 1 km radius). High proportion of cases 65.6% compared with that of control (40.2%) found to have unsafe drinking water storage. Majority of controls (65.2%) compared with case (52.5%) reported to know at least one and more households point-of-use drinking water treatment methods. However, about 80.3% of the cases and 61.5% of controls’ group have ever practiced water treatment at household’s point-of-use.

In the bivariate analysis, the exposure variables that showed significant association with diarrheal-related infant death were households Water Consumption Per Capita per day with less than 25 l/c/day (*P* = 0.031, COR: 0.40, 95% CI: 0.17, 0.92), households water inaccessibility (*P* = 0.028, COR: 0.34, 95% CI: 0.13, 0.89), households with unsafe drinking water storage (*P* < 0.001, COR: 0.35, 95% CI: 0.20, 0.63), households reported as did not practices water treatment at Point-of-use (*P* = 0.007, COR: 0.39, 95% CI: 0.20, 0.77). However, all of these variables as the risk were less likely to be occurred infant death due to diarrhea (see Table 2).

**Table 2:** Frequency distribution and bivariate analysis of drinking water access and use with diarrhea cases and controls in Eastern Ethiopia, 2016-18

Water Supply Characteristics	Case		Control		Crude Odds Ratio (95% CI)	*P*-value
	n	%	n	%		
Households Water Source						
Unimproved	13	21.3	53	21.7	1.03 (0.52, 2.03)	0.945
Improved	48	78.7	191	78.3	1	–
Time to access water						
Above 30 min	21	34.4	91	37.3	1.13 (0.63, 2.04)	0.678
30 min or less	40	65.6	153	62.7	1	–
Distance to access water						
Above 1 km radius	9	14.8	44	18.0	1.27 (0.58, 2.77)	0.546
Within 1 km radius	52	85.2	200	82.0	1	–
Water quantity (Water Consumption Per Capita per day)						
Less than 25 l/c/day	54	88.5	184	75.4	0.40 (0.17, 0.92)*	0.031
25 l/c/day and above	7	11.5	60	24.6	1	–
Households Water Accessibility^a^						
Not accessible	56	91.8	193	79.1	0.34 (0.13, 0.89)*	0.028
Accessible	5	8.2	51	20.9	1	–
Drinking Water Storage						
Unsafe	40	65.6	98	40.2	0.35 (0.20, 0.63)**	0.000
Safe	21	34.4	146	59.8	1	–
Household Point-of-use water treatment knowledge						
Do not know at all	29	47.5	85	34.8	1.70 (0.96, 2.99)	0.068
Knows at least 1 and more methods	32	52.5	159	65.2	1	–
Household Point-of-use water treatment Practices						
Do not treat	49	80.3	150	61.5	0.39 (0.20, 0.77)*	0.007
Treat Water	12	19.7	94	38.5	1	–

*Risk factors significantly associated at *p*-value ≤ 0.05

**Risk factors significantly associated at *p*-value < 0.001

^a^Water Accessible: Households access to at least 25 l per capita per day within 1 km from improved water supply sources

### Risk of sanitation associated with infant’s diarrheal death

*About 65.6% of the cases and 76.2% of controls infants in the households had their own latrine. Nearly a similar proportion of households in cases and controls have practiced open defecation (21.3% for cases and 20.1% for controls). The household’s latrine utilization appears to be 60.7% in cases and 71.3% in the control group. Less than half of the study subject (39.3%) in cases and about 49.6% in controls have found with cleaned latrines. Hand washing facilities near to latrine comprises the less proportion in case (18%) than in the control (50%).* The majority of cases (83.6%) compared with controls (66.4%) in the households found with unimproved sanitation status. Unsafe disposes of child feces in the households appear in large in cases (70.5%) than in control (39.8%). About 54.1% of cases and 79.9% of controls in the households dispose solid wastes in improper way. Majority of cases (82.0%) as compared with controls (57.4%) found with unsafe disposal of liquid wastes by the households.

In the bivariate analysis, households with unimproved sanitation (*P* = 0.011, COR: 0.39, 95% CI: 0.19, 0.80), households with unsafe disposing of child feces (*P* < 0.001, COR: 0.28, 95% CI: 0.15, 0.51), households with improper management of solid waste (*P* < 0.001, COR: 0.21, 95% CI: 0.12, 0.39) were less likely to occur infant death as a result of diarrhea. However, infants in the households who disposed liquid waste unsafely were 3.4 times more likely at risk of diarrheal-associated infant death as compared to infants in the households who properly managed it (*P* = 0.001, COR: 3.38, 95% CI: 1.68, 6.80) (see Table 3).

**Table 3:** Frequency distribution and bivariate analysis of sanitation with diarrhea cases and controls in Eastern Ethiopia, 2016-18

Sanitation Characteristics	Case		Control		Crude Odds Ratio (95% CI)	*P*-value
	n	%	n	%		
Latrine Ownership						
No latrine	21	34.4	58	23.8	0.67 (0.37, 1.22)	0.188
Have Latrine	40	65.6	186	76.2	1	–
Open Defecation Practices						
Yes	13	21.3	49	20.1	0.93 (0.47, 1.85)	0.831
No	48	78.7	195	79.9	1	–
Household Latrine Utilization						
No	24	39.3	70	28.7	0.62 (0.35, 1.11)	0.109
Yes	37	60.7	174	71.3	1	–
Household Latrine Cleanness						
No	18	29.5	64	26.2	1.15 (0.55, 2.39)	0.718
Yes	24	39.3	121	49.6	1	–
Not applicable	19	31.2	59	24.2		
Hand washing Facility near to latrine						
No	31	50.8	63	25.8	0.65 (0.33, 1.28)	0.217
Yes	11	18.0	122	50.0	1	–
Not applicable	19	31.2	59	24.2		
Sanitation Status						
Unimproved sanitation	51	83.6	162	66.4	0.39 (0.19, 0.80)*	0.011
Improved sanitation	10	16.4	82	33.6	1	–
Households Disposing of Child feces						
Unsafe	43	70.5	97	39.8	0.28 (0.15, 0.51)**	0.000
Safe	18	29.5	147	60.2	1	–
Solid waste Management						
Improper management	33	54.1	195	79.9	0.21 (0.12, 0.39)*	0.000
Proper management	28	45.9	49	20.1	1	–
Liquid waste Management						
Improper management	50	82.0	140	57.4	3.38 (1.68, 6.80)*	0.001
Proper management	11	18.0	104	42.6	1	–

*Risk factors significantly associated at *p*-value ≤ 0.05

**Risk factors significantly associated at *p*-value < 0.001

### Risk of hygiene associated with infant’s diarrheal death

The result showed that about 29.5% in case group and 9.8% in controls of the respondents reported as did not washing their hands at any critical time. Handwashing practices was scored less than three critical time of Hand washing, which shows almost similar proportion in both comparative groups *(59.0% for cases and 59.5% for controls). The vast majority of cases (83.6%) compared with controls (32.0%) of households reported not used any agents during handwashing.*

In the bivariate analysis, infants in the households did not practice hand washing in any critical time at all were less likely to occur infant diarrheal death as compared to households practiced three and more critical time of Hand Washing (*P* = 0.025, COR: 0.38, 95% CI: 0.16, 0.89). In contrary, infants in the households practiced less than three critical time of hand washing was three times more likely to have diarrhea related death than households practiced three and more critical time of Hand Washing (*P* = 0.002, COR: 3.02, 95% CI: 1.48, 6.16). The occurrence of diarrhea death among infant’s households who did not use agents (water with soap or ash/abrasives) during hand washing was two times higher than those who used agents (*P* = 0.030, COR: 1.91, 95% CI: 1.07, 3.43) (see Table 4).

**Table 4:** Frequency distribution and bivariate analysis of hygiene characteristics with diarrhea cases and controls in Eastern Ethiopia, 2016-18

Hygiene Characteristics	Case		Control		Crude Odds Ratio (95% CI)	*P*-value
	n	%	n	%		
Critical Time of Hand washing Practices						
Do not practiced hand washing in any critical time	18	29.5	24	9.8	0.38 (0.16, 0.89)*	0.025
Practiced less than three critical time of Hand washing	36	59.0	145	59.5	3.02 (1.48, 6.16)*	0.002
Practiced 3 and more critical time of Hand Washing	7	11.5	75	30.7	1	–
Agents used during Hand washing						
Not used any agents	51	83.6	78	32.0	1.91 (1.07, 3.43)*	0.030
Used (water + soap or ash/abrasives)	10	16.4	166	68.0	1	–

*Risk factors significantly associated at *p*-value ≤ 0.05

### Multivariable’s conditional logistic regression analysis

In the multivariable conditional logistic regression, the risk factors that were significantly associated with infant diarrheal death identified includes age of mother with < 20 years old, unsafe drinking water storage, infants in households without point-of-use water treatment practices, households with unimproved sanitation status, households with unsafe disposing of child feces, households with improper management of solid and liquid waste, households with lesser hand washing practices at critical time.

In this analysis, infants whose age of mother being lower than 20 years old had significant relationship with less likely to occur infant death due to diarrhea as compared to those reference group of age ≥ 35 years (*P* = 0.009, AOR: 0.01, 95% CI: 0.01, 0.47). However, mother’s religious status, mothers and spouse’s level of education did not show statistically significant association with infant’s diarrheal death, particularly after adjustment.

Infants in households with unsafe drinking water storage and households treating their drinking water at point-of-use showed significant association with infant diarrheal death. However, households exposed to unsafe drinking water storage were less likely to be at risk of infant death from diarrhoea as compared to that of safe drinking water storage (*P* = 0.013, AOR: 0.4, 95% CI: 0.18, 0.81). Similarly, those households who did not treat their drinking water at point-of-use were less likely to be at risk of having infant death from diarrheal than those treated their drinking water (*P* = 0.004, AOR: 0.21, 95% CI: 0.08, 0.61).

The occurrence of diarrheal death among infants in households with unimproved sanitation status was less likely than households with those improved sanitation (*P* = 0.050, AOR: 0.36, 95% CI: 0.13, 1.00). Compared to households with the safe disposing of child faeces, those disposing unsafely were found with an increased odds of infant death due to diarrhoea (*P* = 0.014, AOR: 0.34, 95% CI: 0.15, 0.81). Infant diarrheal death were less likely to happen in the households disposing solid wastes improper than those properly managed (*P* = 0.003, AOR: 0.29, 95% CI: 0.13, 0.66).

Households with improper management of liquid waste management found with strong association with more than three times more likely to occur diarrhea-associated infant death as compared to those with proper liquid waste management (*P* = 0.010, AOR: 3.43, 95% CI: 1.34, 8.76). Infants whose mother/caretaker practiced hand washing with less critical time was three times greater risk to infant death from diarrhea than those who had practice more than three critical times of hand washing (*P* = 0.027, AOR: 3.04, 95% CI: 1.13, 8.17) (see Table 5).

**Table 5:** Bivariate and multivariable conditional logistic regression for the risk factors associated with diarrhea-related cases and controls in Eastern Ethiopia, 2016-18

Variables	Case		Control		Odds Ratio (95% CI)	
	n	%	n	%	Crude	Adjusted
Age of the mother						
< 20 Years old	5	8.2	18	7.3	0.14 (0.02, 1.05)	0.05 (0.01, 0.47)*
20-34 Years old	55	90.2	200	82.0	1.01 (0.36, 2.84)	1.59 (0.37, 6.75)
> 35 years old	1	1.6	26	10.7	1	1
Religion						
Muslim	60	98.4	216	88.5	5.65 (0.75, 42.9)	1.00 (0.11, 9.47)
Christian	1	1.6	28	11.5	1	1
Mother’s level of education						
No education	54	88.5	181	74.2	0.37(0.16, 0.86)*	0.50 (0.12, 2.15)
Educated at some level	7	11.5	63	25.8	1	1
Spouse’s level of education						
No education	48	78.7	159	65.2	0.51(0.26, 0.98)*	0.72 (0.21, 2.44)
Educated at Some level of schooling	13	21.3	85	34.8	1	1
Water quantity (Water Consumption Per Capita per day)						
Less than 25 l/c/day	54	88.5	184	75.4	0.40(0.17, 0.92)*	0.21 (0.03, 1.69)
25 l/c/day and above	7	11.5	60	24.6	1	1
Households Water Accessibility						
Not accessible	56	91.8	193	79.1	0.34(0.13, 0.89)*	1.04 (0.10, 10.6)
Accessible	5	8.2	51	20.9	1	1
Drinking Water Storage						
Unsafe	40	65.6	98	40.2	0.35(0.20, 0.63)**	0.38 (0.18, 0.81)*
Safe	21	34.4	146	59.8	1	1
Household Point-of-use water treatment knowledge						
Do not know at all	29	47.5	85	34.8	1.70 (0.96, 2.99)	1.16 (0.45, 2.97)
Knows at least 1 and more methods	32	52.5	159	65.2	1	1
Household Point-of-use water treatment Practices						
Do not treat	49	80.3	150	61.5	0.39(0.20, 0.77)*	0.21 (0.08, 0.61)*
Treat Water	12	19.7	94	38.5	1	1
Latrine Ownership						
No latrine	21	34.4	58	23.8	0.67(0.37, 1.22)	1.47 (0.18, 11.8)
Have Latrine	40	65.6	186	76.2	1	1
Household Latrine Utilization						
No	24	39.3	70	28.7	0.62 (0.35, 1.11)	0.26 (0.04, 1.69)
Yes	37	60.7	174	71.3	1	1
Hand washing Facility near to latrine						
No	31	50.8	63	25.8	0.65 (0.33, 1.28)	0.15 (0.01, 2.36)
Yes	11	18.0	122	50.0	1	1
Sanitation Status						
Unimproved sanitation	51	83.6	162	66.4	0.39(0.19, 0.80)*	0.36 (0.13, 1.00)*
Improved sanitation	10	16.4	82	33.6	1	1
Households Disposing of Child feces						
Unsafe	43	70.5	97	39.8	0.28(0.15, 0.51)**	0.34 (0.15, 0.81)*
Safe	18	29.5	147	60.2	1	1
Solid Waste Management						
Improper management	33	54.1	195	79.9	0.21(0.12, 0.39)*	0.29 (0.13, 0.66)*
Proper management	28	45.9	49	20.1	1	1
Liquid Waste Management						
Improper management	50	82.0	140	57.4	3.38(1.68, 6.80)*	3.43 (1.34, 8.76)*
Proper management	11	18.0	104	42.6	1	1
Critical Time of Hand washing Practices						
Do not practiced in any critical time	18	29.5	24	9.8	0.38(0.16, 0.89)*	0.64 (0.22, 1.87)
Practiced less than three critical times	36	59.0	145	59.5	3.02(1.48, 6.16)*	3.04 (1.13, 8.17)*
Practiced in 3 and more critical time	7	11.5	75	30.7	1	1
Agents used during Hand washing						
Not used any agents	51	83.6	78	32.0	1.91(1.07, 3.43)*	1.92 (0.86, 4.29)
Used (water + soap or ash/abrasives)	10	16.4	166	68.0	1	1

*Risk factors significantly associated at *p*-value ≤ 0.05

**Risk factors significantly associated at *p*-value < 0.001

## Discussion

The present study has attempted to look for possible contributing risk factors for diarrheal-related infant death, predominantly, on Water supply, Sanitation and Hygiene. Although several risk factors were significantly associated with infant’s diarrheal death in the bivariate analysis, some considerable factors that could predispose infants to death were identified after adjusting for confounders. These factors included age of mother with < 20 years old, unsafe drinking water storage, infants in households without point-of-use water treatment practices, unimproved sanitation, unsafe disposing of child feces, households with improper management of solid and liquid waste and households with lesser hand washing practices at critical time.

In our study, infants whose age of mother being lower than 20 years old had significant association with the lower odds of infant’s diarrheal death occurrence as compared to the reference group (age ≥ 35 years). Regardless of the magnitude of risk, this finding is consistence with a case-control study conducted elsewhere, which reported that those infant’s mother with lower maternal age were significantly associated with the risk of diarrhea-associated infant death, particularly among those with normal birth weight [38]. On the other hand, the same study indicated that older maternal age led to a higher chance of diarrhea death among infants with low birth weight. A number of studies have found that lower maternal age tend to have higher risk of mortality in children [39, 40]. This circumstance might be attributed to social and reproductive immaturity. The further likely explanation to this observation is that older mothers are more experienced in childcare and hence there is a possibility in reducing diarrhea-related incidence and death. The lower chance of diarrhea death in infant with younger mother in our study might be the influence of infant biological characteristics such as birth weight or other factors in this maternal age category, which needs further study.

Infants in households with unsafe drinking water storage had significant association with the lower chance of having infant deaths caused by diarrhea. This could be attributed to the unhygienic handling and storage of drinking water that existed in the cases group due to lack of proper and sufficient information on water handling. A study in Benin found that safe water storage had significantly associated with the reduction of diarrhea [41], which in turn lower the risk of death. The national study highlighted that storage water quality issues are a great public health concern in rural Ethiopia [42]. Our study also revealed that death risk of infant diarrhea were significant association with households without point-of-use water treatment practices, which tend to contrary with that of earlier study conducted in southern Brazil [30]. This might be explained by the very small differences among the comparison groups in the household’s water treatment practices. However, other studies suggested as strong significant impact of household water treatment on child survival [43–45]. It is evident that point-of-use water treatment improves the quality of drinking-water by avoiding cross-contamination and prevent diarrheal disease [46, 47], which could considerably reduce risk of mortality [45]. It can be seen from the report that point-of-use water treatment is not widely practiced in Ethiopia [16], posing a health risk of children.

Access to unimproved sanitation has been found to be significantly associated, which had lower odds of diarrhea-related infant mortality as observed in this study. This finding has similarity with another study [48], which designated as improved sanitation significantly associated with lower mortality. Regardless of the association strength, our finding underpins the conclusion made by different studies [31, 48, 49], which indicated as improved sanitation significantly higher association with the reduction of infant mortality. Evidence from the risks quantification study indicated that those countries responsible for the largest declined (13.3%) in the diarrhea mortality rate were reduction in exposure to unsafe sanitation in children [50]. Another study in Egypt also found sanitation to have a more pronounced impact on infants and childhood risk of death from all causes [51]. It can be enlightening that; improved sanitation is fully saved, as it effectively prevents exposure to fecal matters which possibly decrease a major cause of child morbidity and mortality.

Our analyses have been also found that unsafe disposing of child feces had significantly lower risk of infant’s diarrheal death than those households disposing their child feces safely. This observable low risk in the study area might be due to small differences among the two comparison groups on having information and practices of mother/caregivers on save disposal of child feces. Several studies have reported the significant effect of child feces disposal and childhood diarrhea morbidity in Ethiopia [52–54] and elsewhere [55], which could contribute to childhood mortality. Reports indicated that most of the households in high-mortality countries dispose of children’s feces in unsafe manner [56]. It is recognized that, children feces are more dangerous sources of fecal contamination in the household environment, and many cultures however consider the stools of infants are harmless [35, 57]. The findings further indicated that improper management of solid wastes had significantly lower change of infant death due to diarrhea. A number of studies showed that children in households without proper waste collection practices suffer significantly higher rates of diarrhea [18, 58, 59], which are among the main causes of childhood deaths [60]. This is due to the fact that improper disposing of domestic solid waste could be one of the suitable sites for spread of pathogens that can leads to children’s morbidity and followed by mortality.

This study provides notable evidence that improper management of household’s liquid wastes had a positive influence with greater magnitude in infant’s death from diarrhea. The risk of death from diarrhea was more than three times higher in infants living in the households with improper liquid waste disposal than in those disposed in proper way. Reliance on scientific explanation, this might be attributed to the potential source of breading sites for flies which can carry enteric pathogens and mediate a route to contaminate children’s water and food. This result agrees with the general sense that unsafe environment places puts children at risk of death [61]. The strong significant differences observed in this study might be attribute to households were not well aware with the effect of domestic liquid waste disposal on health outcome of children.

Despite the fact of human hands is one of the main vehicles for transmitting diarrhea disease, the role of poor handwashing practices at critical time as a risk factor had strong relationship with infant death from diarrhea in the study area. Our study demonstrated that those self-reported household’s practiced handwashing in less occasions (One to two critical times’) increases risk of infant deaths from diarrhea by 3-fold greater compared with those washed their hands at three and more critical times. Regardless of its magnitude, different studies indicated the significant link between handwashing practices and diarrhea morbidity and mortality [62–64]. The handwashing practices at critical time mainly in mothers of children is poor according to inland study [65]. Evidences indicated that handwashing at critical times reduce diarrhea rated by almost 40% [66], which could be resulted significant reduction in mortality.

### Strength and limitation

The strengths of this study include as it used nested case-control study design which is valid and efficient design and can minimized both selection and recall bias. Despite such studies is available insufficiently at population level, in particular, this study could provide useful information for building evidence based health policy, better insights in planning the best solutions, and generate ideas for further research.

This study has limitations as respondents might not give their exact observable fact towards some given questions, which leads to social desirability biases. This could be minimized by providing training to data collectors and conducted pre-test before actual data collection launched. A very shortage of previous similar studies was made comparison difficult.

## Conclusion

In conclusion, this study finding pointed out the risk factors that were statistically significant associated with less likely to occurred diarrhea-related infant death include; age of mother with < 20 years old, unsafe drinking water storage, infants in households without point-of-use water treatment practices, households with unimproved sanitation status, unsafe disposing of child feces and improper management of solid waste. On the other hand, households with improper management of liquid waste and household’s practiced handwashing in fewer occasions (One to two critical times’) were significantly associated with more likely to contribute lost of infant life due to diarrhea. The significant link of such risk factors could be one of the reasons for the contribution of uppermost death level of infants as the result of diarrhea. Therefore, efforts should be made to ensure intervention taking such risk factors into consideration which eventually can turn-down the consequence of the highest reported number of diarrhea-related infant death.

## Data Availability

We confirm that all the relevant data are fully available. The dataset are accessible by contacting the corresponding author and provided upon a reasonable request.

## Abbreviations

AOR: Adjusted Odds Ratio
CI: Confidence Interval
COR: Crude Odds Ratio
CSPro: Census Statistics Program
EIWR: Ethiopian Institute of Water Resources
ETB: Ethiopian Birr
L/c/d: Liters per capital per day
SD: Standard Deviation
SPSS: Statistical package for Social Sciences
WHO: World Health Organization
